# Prognostic value of proadrenomedullin in patients with COVID-19 pneumonia

**DOI:** 10.3389/fmed.2022.961071

**Published:** 2022-08-22

**Authors:** Aleksandr A. Astapovskii, Vladimir N. Drozdov, Evgenia V. Shikh, George G. Melkonyan, Zhanna M. Sizova, Valeria L. Zakharova, Natalia N. Shindryaeva, Natalia I. Lapidus

**Affiliations:** ^1^Department of Clinical Pharmacology and Propedeutics of Internal Diseases, First Moscow State Medical University Named After I.M. Sechenov, Moscow, Russia; ^2^Department of Health, Moscow Veterans Hospital No3, Moscow, Russia; ^3^Department of Social Expertise, Urgent and Outpatient Therapy, First Moscow State Medical University Named After I.M. Sechenov, Moscow, Russia; ^4^Department of Nervous Diseases and Neurosurgery, First Moscow State Medical University Named After I.M. Sechenov, Moscow, Russia

**Keywords:** COVID-19, proadrenomedullin, coronavirus, biomarkers, nephrology

## Abstract

**Objective:**

The aim of the study was to assess the role of mid-regional proadrenomedullin (MR-proADM) in comparison with routine laboratory tests in patients with COVID-19.

**Materials and methods:**

140 hospitalized patients aged 18 and older with COVID-19 pneumonia were included in prospective single-center study. Routine analyses were performed, and MR-proADM level was measured within the first and the third hospital days using Human MR pro-ADM (Mid-regional pro-adrenomedullin) ELISA Kit with a sensitivity of 0.469 pmol/L (immunofluorescence assay). National Early Warning Score (NEWS) was used for primary assessment of the disease severity. According to disease outcome the patients were divided into two groups: discharged patients (*n* = 110, 78.6%) and deceased patients (*n* = 30, 21.4%). Results: The groups had no statistically significant difference in sex, comorbidity, body temperature, oxygen saturation level, heart rate, respiratory rate, and C-reactive protein (CRP) level and procalcitonin (PCT). The deceased patients had statistically significant difference in age (median, 76 years; interquartile range, 73.2–78.2 vs. median, 66 years; interquartile range, 62–67; *p* < 0.0001), NEWS value (median, 5; interquartile range, 3–8 vs. median, 2; interquartile range, 0–6; *p* <0.05), hospitalization period (median, 17; interquartile range, 7–35 vs. median, 6; interquartile range, 3–14), quantitative CT extent of lung damage > 50% [*n* = 26 (86.7%) vs. *n* = 9 (8.2%) *p* < 0.0001], level of leukocytes (median, 11.4 ×109/L; interquartile range, 6.2–15.5 vs. median, 5.3 ×109/L; interquartile range, 4.7–6.4; *p* = 0.003), level of neutrophils (median, 80.9%; interquartile range, 73.6–88.6 vs. median, 72.6%; interquartile range, 68.7–76.9; *p* = 0.027), level of MR-proADM at the first hospital day (median, 828.6 pmol/L; interquartile range, 586.4–1,184.6 vs. median, 492.6 pmol/L; interquartile range, 352.9–712.2; *p* = 0.02), and level of MR-proADM at the third hospital day (median, 1,855.2 pmol/L; interquartile range, 1,078.4–2,596.5 vs. median, 270.7 pmol/L; interquartile range, 155.06–427.1).

**Conclusion:**

Mid-regional proadrenomedullin has a higher prognostic value in patients with COVID-19 in comparison with routine inflammatory markers (leukocyte and neutrophils levels, CRP, and PCT) and NEWS.

## Introduction

In December 2019, an outbreak of pneumonia caused by a new coronavirus named SARS-CoV-2 occurred in Wuhan, Hubei Province, China (1). Toward December 2021, more than 260 million disease cases and 5 million fatal outcomes were confirmed worldwide, according to the World Health Organization (2).

The new coronavirus infection, which is called COVID-19 (an acronym that stands for coronavirus disease of 2019), is a systemic inflammatory disease which mostly affects the respiratory system. Ingress of infection occurs though type 2 angitensin-converting enzyme, which is widely expressed in a variety of organs and tissues, including the lungs, heart, kidneys, intestines, and endothelial cells (3). The disease course ranges from asymptomatic to the development of severe pneumonia and acute respiratory distress syndrome with a high mortality rate (4–13%) (4). A distinctive feature of SARS-CoV-2 infection is the immune system activation which can lead to an uncontrolled generalized inflammatory response, the so-called “cytokine storm” (5).

Fast growing incidence of the disease and limited health care resources require quick and objective stratification of patients based on the disease severity and prognostic risk. Nowadays, there are different biomarkers and medical scores which allow to assess disease severity and choose appropriate medical care (6). Among these are IL-6, C-reactive protein (CRP), and procalcitonin (PCT). The levels of these biomarkers are increased in patients with COVID-19 (7, 8). Timely and objective stratification of patients is necessary, as it allows to balance the healthcare system budget, reduce unjustified expenses, and distribute patients according to risk groups to provide proper medical assistance.

In the past few years, new biomarkers with high prognostic value have appeared in clinical practice. One of these biomarkers is adrenomedullin.

Adrenomedullin is a multifunctional peptide, or hormokine, consisting of 52 amino acids. It was isolated by Japanese scientists K. Kitamura et al. in 1993 from human pheochromocytoma. It belongs to the family of vasoactive peptide hormones associated with the calcitonin gene (9). The term “hormokine” refers to the ability of certain hormones to act like cytokines. Adrenomedullin has a strong vasodilator effect. It reduces vascular resistance indirectly *via* increased synthesis of NO by endothelial cells, increased concentration of cAMP and calcium (10). Adrenomedullin reduces vascular permeability and the formation of pro-inflammatory cytokines, also it leads to increase in diuresis and natriuresis (11, 12). Adrenomedullin has a protective effect in cases of impaired tissue microcirculation and prevents the development of tissue hypoxia (13). Increased serum level of adrenomedullin indicates the development of organ failure, which allows to use it as an early biomarker of various pathological conditions (cardiovascular and respiratory diseases, sepsis) for diagnostics and prognostics (14–16).

The reliability and accuracy of adrenomedullin level determination in the blood are limited due to fast clearance (about 22 mins), rapid degradation by proteases, and plasma protein binding (17, 18). The measurement of mid-regional proadrenomedullin (MR-proADM) provides a solution to this problem. MR-proADM is more stable molecule than adrenomedullin and has a longer half-life. It is cleaved from the precursor molecule on a 1:1 ratio and is not degraded by proteases, because it is supposed to be non-functional metabolic product (18).

Several studies have demonstrated that MR-proADM has a higher prognostic value than other biomarkers such as PCT and CRP (19).

The aim of the study is to assess the prognostic value of MR-proADM and compare it with other routine clinical and laboratory analyses in patients with COVID-19.

## Materials and methods

### Study design and population

We conducted a single*-*center prospective study. 140 patients over the age of 18 with COVID-19 pneumonia were enrolled in this study. They were admitted to the State Budgetary Institution of Healthcare of the City of Moscow "City Clinical Hospital No. 4 of the Department of Moscow Healthcare Department.” The patients were enrolled in the study between June 2021 and September 2021. The study protocol was approved by the local ethics committee of Federal State Autonomous Educational Institution of Higher Education I.M. Sechenov First Moscow State Medical University of the Ministry of Healthcare of the Russian Federation (Sechenov University) and was carried out in accordance with Declaration of Helsinki.

All patients enrolled in the study had COVID-19 pneumonia, confirmed by clinical and diagnostic data, including positive COVID-19 PCR test. Computed tomography (CT) of the chest was performed for all patients on the first day of hospitalization. The degree of lung was assessed according to the methodological recommendations: Radiation diagnosis of coronavarius disease (COVID-19): organization, methodology, interpretation results (20) Patients received medical treatment according to Interim Guidelines for the prevention, diagnosis, and treatment of new coronavirus infection (COVID-19), version 11 (05/07/2021) (21). National Early Warning Score (NEWS) was used for initial assessment of the disease severity. The score includes assessment of the respiratory system function (respiratory rate, oxygen saturation), the need for supplemental oxygen, body temperature, systolic blood pressure, heart rate, and level of consciousness (22). All patients enrolled in the study were divided into two groups depending on the disease outcome: discharged patients (*n* = 110) and deceased patients (*n* = 30).

### Data collection

Data were collected from electronic medical records and laboratory information systems. Required data included demographic variables, comorbidities, and laboratory test results.

### Blood draw and laboratory analysis

All the blood samples were taken from the patients within 1 h after hospitalization. Blood samples for measuring MR-proADM were collected in EDTA tubes on the first and third days of hospitalization. The blood samples were centrifuged for 10 mins, then the plasma was frozen and stored at−80 C until testing. MR-proADM concentration was measured in thawed plasma samples using Human MR pro-ADM (Mid-regional pro-adrenomedullin) ELISA Kit (Fine Biotech, China), with a sensitivity of 0.469 pmol/ml (immunofluorescence assay).

### Statistical analysis

Statistical processing of the results was carried out using Medcalc^®^ version 19.8. All the data were presented as absolute values, median and interquartile range. Mann-Whitney *U*-test was used for the data comparison. *P*-values < 0.05 were statistically significant. We used ROC-analysis to identify prognostic values of studied predictors. Values with statistically significant difference between the groups of discharged and deceased patients were included in the analysis.

## Results

Tables 1, 2 illustrate clinical and demographic characteristics of patients and inflammatory markers, respectively. The groups had no statistically significant difference in sex, comorbidities, body temperature, oxygen saturation level, heart rate, respiratory rate, C-reactive protein and procalcitonin levels. The deceased patients had a statistically significant increase in age, NEWS value, hospitalization period, quantitative CT extent of lung damage, leukocyte and neutrophil counts, levels of MR-proADM on the first and the third day of the hospitalization.

**Table 1:** Clinical and demographic characteristics of patients.

**Characteristic**	**Discharged (110)**	**Deceased (30)**	***p***
Number of patients, *n* (%)	110 (78.6)	30 (21.4)	
Age	66 (62–67)	76 (73.2–78.2)	<0.0001
**Sex**			
Males, *n* (%)	38 (79)	10 (21)	0.9017
Females, *n* (%)	72 (78)	20 (22)	
Bed day	6 (3–14)	17 (7–35)	<0.0001
**Quantitative CT extent of lung damage**			
From 0 to 25%	56 (50.9%)	0 (0%)	<0.0001
From 25 to 50%	45 (40.9%)	4 (13.3%)	0.03
From 50 to 75%	9 (8.2%)	19 (63.3%)	<0.0001
Over 75%	0 (0%)	7 (23.4%)	<0.0001
Body temperature, C	38.1(37.8–38.4)	38.0(37.8–38.2)	0.139
SpO_2_, %	93 (82–97)	89 (78–93)	0.865
Respiratory rate	21 (18–24)	22 (20–24)	0.997
Heart rate	80 (69–100)	80 (70–114)	0.178
NEWS	2 (0–6)	5 (3–8)	<0.0001
**Co-morbidities**			
Hypertension	66 (60%)	23 (76.6%)	0.267
Diabetes mellitus	14 (12.7%)	10 (33.3%)	0.446
Oncological diseases	5 (4.5%)	2 (6.7%)	0.628
Chronic heart failure	20 (18.2%)	11 (36.6%)	0.732
Chronic renal insufficiency	5 (4.5%)	5 (16.6%)	0.835
Chronic respiratory disease	6 (5.5%)	4 (13.3%)	0.132
Other	11 (11%)	7 (23.3%)	0.329

CT, computed tomography; SpO_2_, oxygen saturation of the blood.

**Table 2:** Inflammatory markers.

**Characteristic**	**Discharged**	**Deceased**	***p***
Leucocytes, 1 × 10^9^/L	5.3 (4.7–6.4)	11.4 (6.2–15.5)	0.003
Neutrophils, %	72.6 (68.7–76.9)	80.9 (73.6–88.6)	0.027
CRP, mg/L	37.5 (20.2–57.8)	25.6 (14.3–59.1)	0.505
PCT, ng/ml	0.079 (0.054–0.0104)	0.092 (0.077–0.22)	0.156
1st day-MR-proADM, pmol/L	492.6 (352.9–712.2)	828.6 (586.4–1,184.6)	0.02
3rd day-MR-proADM, pmol/L	270.7 (155.06–427.1)	1,855.2 (1,078.4–2,596.5)	<0.0001

CRP, C-reactive protein; PCT, procalcitonin; MR-proADM, mid-regional pro-adrenomedullin.

**Figure 1:**
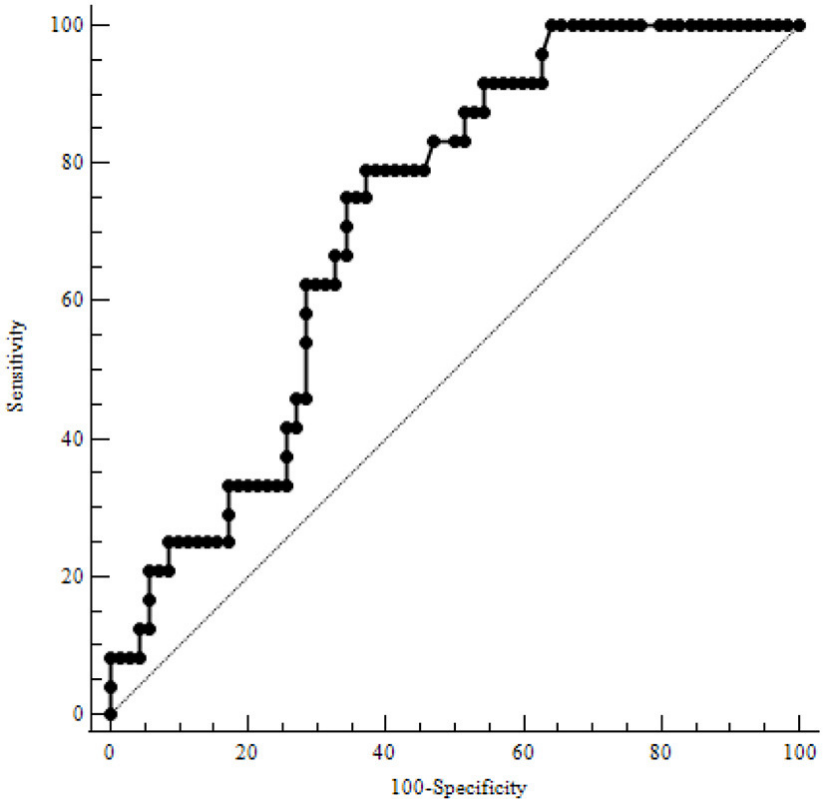
ROC-analysis of MR-proADM level at the 1st day of hospitalization. MR-proADM—mid-regional pro-adrenomedullin.

**Figure 2:**
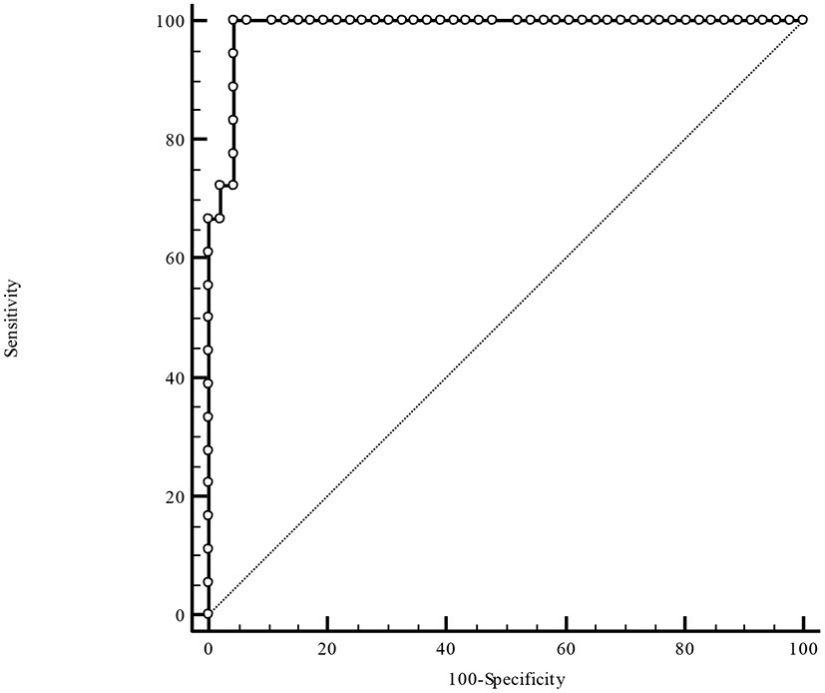
ROC-analysis of MR-proADM level at the 3rd day of hospitalization. MR-proADM—mid-regional pro-adrenomedullin.

Table 3 illustrates the results of ROC-analysis of association between proadrenomedullin concentration, CRP and PCT levels, and poor outcome in patients with COVID-19. The data suggest that MR-proADM measured on the first and third days of hospitalization had the highest prognostic value in comparison with other inflammatory markers (Figures 1, 2). Among the studied biomarkers, the biggest AUC was observed in MR-proADM. 1st day-MR-proADM: AUC ROC = 0.72 (0.57–0.84); cut-off = 500 pmol/L; sensitivity 79.2%, specificity 69.2%. The 3rd day-MR-proADM: AUC ROC = 0.98 (0.86–1.0); cut-off = 700 pmol/L; sensitivity 100%, specificity 95.6%. The relative risk (RR) of poor outcome for MR-proADM concentration higher than 500 pmol/L was 2.8 (95% CI 1.7–4.6; sensitivity 83.3%, specificity 72.7%), while RR for MR-proADM concentration higher than 700 pmol/L was 21 (95% CI 3.1–142.2; sensitivity 100%, specificity 91.3%). We also noted that the MR-proADM level significantly decreased by the 3rd day of hospitalization in the discharged group (*p* = 0.0024), while it significantly increased in the deceased group (*p* = 0.0077).

**Table 3:** ROC-analysis results of poor outcome predictors.

**Characteristic**	**AUC ROC (95% CI)**	**Cut-off**	**Sensitivity, %**	**Specificity, %**	***p***
CRP, mg/L	0.55 (0.41–0.77)	45	73.3	45.6	0.48
PCT ng/ml	0.62 (0.49–0.73)	0.07	80%	48.2%	0.14
1st day-MR-proADM, pmol/L	0.72 (0.57–0.84)	500	79.2%	62.9%	0.004
3rd day-MR-proADM, pmol/L	0.98 (0.86–1)	700	100%	95.6%	<0.0001

CRP, C-reactive protein; PCT, procalcitonin; MR-proADM, mid-regional pro-adrenomedullin.

## Discussion

In this study, we aimed to evaluate the prognostic value of MR-proADM in COVID-19 patients. It was shown that the level of MR-proADM on days 1 and 3 of hospitalization in deceased patients was 1.7 and 6.9 times higher, respectively. In addition, we found a further significant increase in MR-proADM level in deceased patients by the third day of hospitalization, while in discharged patients its level significantly decreased. The results suggest that MR-proADM measurement in dynamics may be useful for early risk stratification (upon admission to hospital) and for monitoring COVID-19 patients. The results demonstrated that the high prognostic value of MR-proADM can rule out nosocomial mortality in patients with low MR-proADM levels, which could potentially help improve patient triage.

COVID-19 pandemic has affected hospitals resources; therefore, optimization *of* hospital admission is required to provide medical care to the patients with severe disease course. Thus, stratification of patients is essential, because it helps to choose an appropriate extent of medical care. That is why, current researches are aimed at finding the most reliable predictors.

For example, scores CURB-65 and PSI were developed to assess the severity of community-acquired pneumonia and to predict mortality, as well as SOFA and qSOFA were developed for predicting mortality in ICU patients (23, 24). However, these scales are not perfect due to it requires measurement of plenty clinical and laboratory parameters, which cannot always be performed even within the first hospitalization day, as well as it has lack of prognostic value.

MR-proADM is a promising biomarker for predicting outcomes in patients with different pathologies. In 2006, it was demonstrated that the level of MR-proADM was significantly higher in patients with community-acquired pneumonia with poor outcomes; C-reactive protein did not show similar pattern (25). We had comparable results in our research.

The prognostic scores for patients with community-acquired pneumonia or sepsis do not have strong predictive value, especially in young patients. One study showed that proadrenomedullin measurement together with pneumonia severity assessment according to SCAP score had significantly increased accuracy of disease course prognosing (26). Another study demonstrated that proadrenomedullin had the best predictive accuracy of 28-day mortality in patients with sepsis or septic shock in comparison with C-reactive protein, procalcitonin, and lactate. Also, proadrenomedullin measurement increases sensitivity and specificity of the SOFA score (27).

Nowadays, proadrenomedullin is actively being studied as a prognostic biomarker in patients with novel coronavirus disease (COVID-19). Gregoriano et al. were among the first to demonstrate proadrenomedullin as a predictor of in-hospital mortality among patients with COVID-19 due to its accuracy. The authors noted that elevated levels of proadrenomedullin on admission and during hospital stay were independently associated with in-hospital mortality and may allow better risk stratification and, in particular, rule-out of fatal outcome, in COVID-19 patients (28).

In another similar study, the authors also concluded that elevated levels of proadrenomedullin were significantly associated with the high risk of in-hospital mortality. The ROC analysis demonstrated that the sensitivity and specificity of proadrenomedullin as a predictor of adverse outcome was 90 and 95%, respectively, which is consistent with our data (29).

Our study presents some limitations. Firstly, the number of analyzed cases was small due to the single center design of this study. Secondly, the level of proadrenomedullin was assessed over time, while the levels of C-reactive protein and procalcitonin were assessed on the first day of hospitalization, which could affect the results of the ROC-analysis. Despite these, our findings suggests that MR-proADM can potentially assist in identifying the most severe cases and clinical decision makings in COVID-19.

## Conclusion

In terms of the pandemics, biomarkers measurement may improve stratification of patients according to the disease severity and expected outcomes. In addition, accurate stratification helps to reduce hospital load, leads to the optimal use of the health care resources without affecting the quality of medical care. In our study, mid-regional proadrenomedullin has the highest prognostic value as a predictor of poor outcome in patients with COVID-19, compared to leukocytes and neutrophils levels, C-reactive protein and procalcitonin concentrations.

## Data availability statement

The raw data supporting the conclusions of this article will be made available by the authors, without undue reservation.

## Author contributions

All authors listed have made a substantial, direct, and intellectual contribution to the work and approved it for publication.

## Conflict of interest

The authors declare that the research was conducted in the absence of any commercial or financial relationships that could be construed as a potential conflict of interest.

## Publisher's note

All claims expressed in this article are solely those of the authors and do not necessarily represent those of their affiliated organizations, or those of the publisher, the editors and the reviewers. Any product that may be evaluated in this article, or claim that may be made by its manufacturer, is not guaranteed or endorsed by the publisher.

## Abbreviations

MR-proADM: midregional proadrenomedullin
NEWS: National Early Warning Score
IL: interleukin
CRP: C-reactive protein
PCT: procalcitonin
PCR: polymerase chain reaction
CT: computed tomography
EDTA: ethylenediaminetetraacetic acid
AUC: area under the curve
ICU: intensive care unit.
